# A Guide to Scientific Crowdfunding

**DOI:** 10.1371/journal.pbio.1002373

**Published:** 2016-02-17

**Authors:** Julien Vachelard, Thaise Gambarra-Soares, Gabriela Augustini, Pablo Riul, Vinicius Maracaja-Coutinho

**Affiliations:** 1 Beagle Bioinformatics, Santiago, Chile; 2 Dodo Funding, Santiago, Chile; 3 Facultad de Arquitectura, Diseño y Estudios Urbanos, Pontifícia Universidad Católica de Chile, Santiago, Chile; 4 Olabi Makerspace, Rio de Janeiro, Brazil; 5 Departamento de Engenharia e Meio Ambiente, CCAE, Universidade Federal da Paraíba, Rio Tinto, Brazil; 6 Centro de Genómica y Bioinformática, Facultad de Ciencias, Universidad Mayor, Santiago, Chile; 7 Instituto Vandique, João Pessoa, Brazil

## Abstract

Crowdfunding represents an attractive new option for funding research projects, especially for students and early-career scientists or in the absence of governmental aid in some countries. The number of successful science-related crowdfunding campaigns is growing, which demonstrates the public’s willingness to support and participate in scientific projects. Putting together a crowdfunding campaign is not trivial, however, so here is a guide to help you make yours a success.

## Abstract 2

A new way of funding research has recently emerged, potentially democratizing the way science is done. This article provides a guide to increasing the odds of financing your research through crowdfunding.

## Introduction

In the past 20 years, the internet and social media have brought about dramatic changes in scientific practice towards a more collaborative and connected approach with the general public and citizen scientists. This changing paradigm is also opening new ways to raise funds for research. Scientists (especially in their early careers), students, and researchers in developing countries (where research funding is scarcer), have started to seek funding through “crowdfunding” [1]. Crowdfunding involves asking for small sums of money from a large number of individuals. Most of the time, donations are rewarded with symbolic gifts (e.g., T-shirts, mugs, mention in a paper). Usually, the project is uploaded among others on an internet platform that enables project owners to launch their campaigns. Crowdfunding campaigns take the form of marketing and communication campaigns, in which the project owner tries to reach as many backers as possible through social networks and media coverage. So, crowdfunding enables people outside academia to engage with science on a global scale. It can also be an alternative or additional source of funding, given the difficulties of raising funds in the traditional science funding system. Although crowdfunding cannot be the solution to the current funding crisis [2], it can be a viable alternative for certain types of research projects.

**Fig 1:**
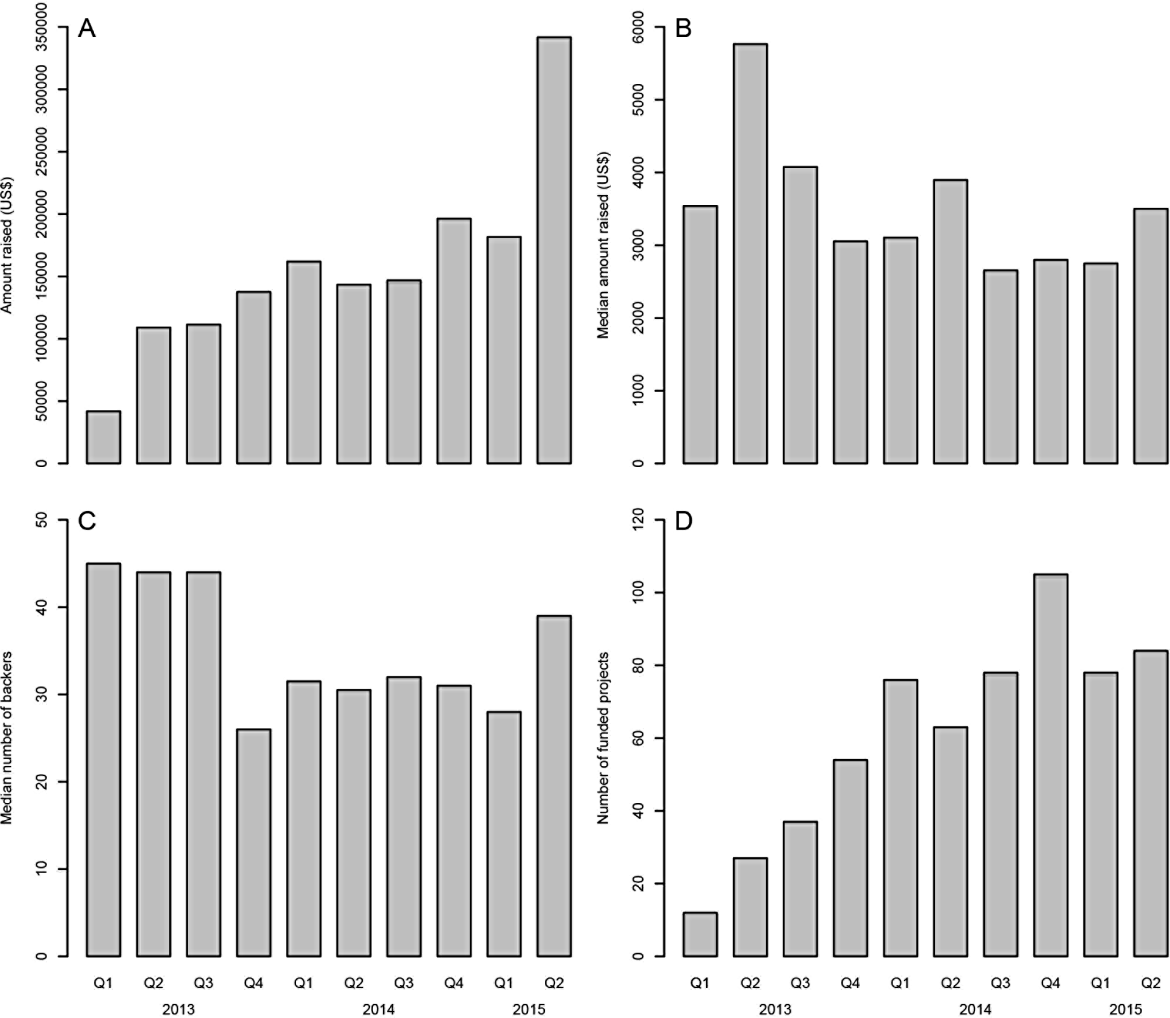
Successful crowdfunding campaigns on Experiment.com by quarter from January 2013 to July 2015. (A) Total amount raised, (B) median amount raised, (C) median number of backers, and (D) number of funded projects.

The number of successful science-related crowdfunding campaigns is growing, and these campaigns demonstrate the public’s willingness to support and participate in scientific projects [3–5]. Data from Experiment.com (a platform dedicated to scientific crowdfunding) suggests a trend of increase in total amount raised and number of funded projects per quarter from 2013 to 2015, although the median amount raised and median number of backers remained relatively constant (Fig 1). SciFund Challenge—a nonprofit organization seeking to connect scientists with society through crowdfunding—achieved a success rate of almost 70% of funded projects in its latest round (February–March 2014) by supporting and guiding scientific crowdfunding campaigns on the general crowdfunding platform RocketHub [6]. This represented an increase of approximately 50% since its first round in November–December 2011.

Crowdfunding is still at an early stage, and there are no publicly available data allowing one to reliably estimate the odds and rates of success related to scientific crowdfunding. It is also difficult to measure the time and cost involved in a crowdfunding campaign versus a traditional grant application. It should be clear to any scientist attempting to crowdfund a project that the communication efforts required can be quite taxing and that a fair amount of preparation is needed in order to have a chance at success [4,5,7]. However, the benefits and the experience itself can be much more rewarding than a grant application, since you might meet new collaborators and private investors, build unexpected bridges for your research, and/or connect with the general public in a direct and interesting way. Pioneering crowdfunding efforts provide very useful pointers to best practices for those who wish to try their hand at crowdfunding their projects [4,5,7]. In this article, we provide a general guide for crowdfunding, explaining what is involved in making your campaign a success.

### Who Should Pursue a Crowdfunding Campaign?

Anybody can pursue a crowdfunding campaign. However, such a campaign is not a miraculous solution, and it requires a significant investment of time and effort in science outreach. The likelihood of successfully fundraising through community participation is directly dependent on the effort you invest in communicating about your research with the public. It would be a mistake to assume that all you have to do is put the project on the Web, then sit back and wait for easy money. A crowdfunding campaign is “a constant marathon of social media networking”, as Li and Pryer note [7].

Although setting up a crowdfunding campaign can be as time-consuming as a traditional grant application, it is a promising alternative, particularly for researchers at the beginning of their careers (i.e., undergraduate and graduate students, post-doctoral researchers, and early-career scientists) who are unable to compete for funding with renowned scientists. Crowdfunding is also an alternative for researchers living in countries where funding is scarce, and it has the potential to change the local research landscape.

### Involve As Many People As You Can

The success of a crowdfunding campaign strongly depends on the network of the project owner [4,5,7–9]. The use of online social media, such as Facebook, LinkedIn, and Twitter, is essential. You have to mobilize all your connections before launching the project on a crowdfunding platform. The owner of the Crowd4Discovery campaign estimated that 60% of the backers came from his Twitter and Facebook followers and friends [4]. So, if you do not have a strong network, start building one as soon as possible because it can be quite a long process.

You should also consider other tools, such as general forums and hubs like Reddit (www.reddit.com) and blogs. By using Reddit, for example, Li and Pryer substantially increased the number of visitors to their campaign to sequence the *Azolla* fern genome [7]. They created an “Ask Me Anything” thread, in which anyone could ask them any question related to *Azolla*, ferns, and plants during one afternoon. Spreading the word in this way may even lead to an influential blogger mentioning your project.

Do not forget the traditional “offline” social networks: your family, friends, and colleagues. During the early stages of your campaign, these networks are more important than online ones for a simple reason: trust. They know you and are willing to help you. You have to build a core of followers and supporters around your project. Doing so shows that people trust you and, consequently, your online network will be more willing to help you.

You can also increase your network through events and talks about your research. Coworking spaces, makerspaces, hackerspaces, startup incubators, and entrepreneurship hubs are usually open to listening to new projects. Engaging with the local scene and participating in groups and activities in these kinds of spaces is a way to meet people that can expand your network of potential backers in a short amount of time.

### Be Passionate about Your Project

You have to show that your project is worthy of attention in order to convince people to share the idea, be part of the project, and ultimately fund it. Crowdfunding is as much about passion as it is about trust. For example, take a look at the ARKYD project video, made for one of the most successful science-related campaigns, which raised more than 1.5 million dollars to launch a publicly accessible telescope [10]. The common factor between all the participants in this video is their strong enthusiasm about the project.

### Communicate Clearly and Engagingly

In general, people do not have a lot of free time, and their attention span on the Internet is very limited. Remember that you will be competing for attention with a picture of a kitten on Facebook or the latest Rihanna clip on YouTube. Although some of your backers will be scientists themselves, they will be a minority. You have to be clear and engaging, go beyond the technical aspects of your project, and present the “whys” of your work: sociological, historical, economic and/or environmental perspectives.

The campaign “Can anle138b delay the onset of genetic prion disease?” is a good example of how to manage a crowdfunding campaign on a complex research project [11]. Their campaign video explains the science in a pedagogical way in the first minute. The remaining of the video focuses on why the project organizers are conducting this research rather than how.

### Tell People about Your Project, but Above All Show Them

Campaigns with pictures and videos are significantly more successful than ones without. People must *like* your project more than they have to *understand* it, and the power of images to achieve this bond is much greater than that of text. Good examples are the campaigns developed by the already-mentioned ARKYD project [10] and uBiome, a project to study the human gut flora [12].

Here are a few simple rules for producing a homemade video:

- Keep it short. The first 30 seconds are the most important, so be sure that this is the most powerful part of your video.
- Keep it “clean.” Do not tire viewers with poor sound, bad lighting, or wobbly images.
- Provide clear answers to the following questions: why are you asking money from the crowd and what is the big picture behind your project?

### Do Not Ask for the Moon

Crowdfunding is not ready (yet) to raise funds in science for very expensive projects and cannot replace traditional grants [13]. Therefore, it is important to be realistic about the amount you can ask and the scope of your project. Most crowdfunding platforms dedicated to science operate in an all-or-nothing or in a flexible model. The former means that you only get funded if you reach the total amount you are requesting. Thus, asking for too much can be a way to end up with nothing. The flexible model enables you to keep every dollar raised even if you do not reach your goal. If you think that the sum you need for your project is paramount, try to divide it into smaller meaningful projects, but keep in mind that you must show your audience the big picture.

A blog post on the funding goals of the most successful projects on the platform Experiment.com [14] provides a good indicator of what you can expect to raise for a scientific project. The maximum amount raised by a unique project on this platform in 2015 was US$139,951, with a general average of US$6,306 and a median of US$3,585 (data as of July 16, 2015). However, this is not the limit for scientific crowdfunding in general. Between July 2011 and March 2014, the SciFund Challenge on another platform, RocketHub, has seen around 170 science projects fundraising through the crowd. There, the maximum amount raised by successful campaigns was US$10,000 [6]. Science crowdfunding is evolving, and it is already achieving bigger sums. Some successful science-related campaigns have achieved over US$100,000. uBiome [12] and American Gut [15], both projects studying the human gut flora, reached more than US$350,000. The ARKYD telescope raised over US$1.5 million [10]. Some successful computational-biology–related campaigns also surpassed US$100,000 in donations: OpenWorm (US$121,076), a digital simulation of the entire *Caenorhabditis elegans* organism [16]; OpenTrons (US$126,694), automated robots for molecular biology laboratories [17]; and Parallella (US$898,921), a low-cost supercomputer [18].

### Be Precise and Rigorous about How You Will Be Using the Money

You have to be quite precise about the amount of money you need. Once again, it is all about trust, and you must be able to tell people precisely which part of their money will be used for which part of your project (including the costs of the rewards and the crowdfunding campaign itself). It is a real asset to present a chart showing how the budget is divided. It is also important to make it clear that you are dealing with experimental efforts, and that it could take months or even years before a conclusive answer and positive outcome is obtained. In the worst-case scenario, backers should be aware that you might not reach any conclusion.

### Give Meaningful Rewards

Scientific crowdfunding is a little bit different from classic crowdfunding in this regard, especially because in some fields you will not necessarily have a product to give to your backers. However, a reward can also take the form of an opportunity. People are not on a scientific crowdfunding platform to get a pre-released version of a product, as commonly happens on other platforms. They actually have a more idealistic view and want to enable you to conduct your research and develop your ideas. If it is possible, reward them with meaningful and creative gifts. For example, a day in the field, in the lab with you, or a mention in your paper or webpage acknowledgments. If your project leads to a product, provide a first sample of your prototype as a reward.

### Update, Update, Update

People on a scientific crowdfunding platform are there because they share an interest in your research. The least you can do is give them frequent updates on the progress of the project. The public must feel that they are a part of the project. You should send messages and post updates as often as you can about how the campaign is going and how you appreciate their support. It will create engagement and show your commitment to the project.

### Expect Some Negative Reactions

You will surely have some supportive feedback after sharing your campaign. Sadly, you will also have some negative comments in social media. You have to take into account the constructive ones, as they can help you with the progress of your campaign and your project, and just ignore or erase the purely negative and offensive ones.

### It Is Not All about Money

We hope that if you followed the previous recommendations, you will not have to read this part. However, sadly, similarly to a grant application, crowdfunding campaigns can fail. It is not the ideas, the research, or the technology you are developing that have failed. It is only your campaign. The reasons for failure are almost always extrinsic, the main one being the failure to reach a small percentage of the total sum requested quickly enough “to get the ball rolling” [19].

However, can we really call it failure if a project doesn’t succeed in reaching its fundraising target? After a campaign, you have improved your network, might have new collaborators, and might have obtained funds from investors. More generally, you were given the possibility to present your research widely to total strangers all over the world and discuss it with them. You were, thus, improving your ability to reach out and communicate your ideas to the public.

One example of these side benefits can be found in the *Azolla* fern campaign [7]. As stated by the project owners, *“*Halfway through the campaign, and having reached one-third of our funding goal, our journey took an unexpected turn: BGI (formerly the Beijing Genomics Institute, but now based in Shenzhen) fully backed our project. BGI did not pledge actual money; instead they offered to fulfill all our sequencing needs free of charge.”

Money is only one of the several benefits of a crowdfunding campaign. Still, you need the money to fund your research, so consider going back to the drawing board, review what went wrong, try to learn from the experience of others as much as you can, and with the improved network and experience you gained, the next campaign will likely be a success.

### A Call for Openness

Crowdfunding seems to have significant potential. However, to fully assess this potential, we would need access to data that are unfortunately not publicly available at the moment. We urge platform owners to make more effort to enable open access to their data. By transparently publishing failure rates, sums asked per project, and sums actually raised, platforms owners would not be hurting their businesses. Instead, they would greatly help the scientific community decide when and in which cases it is worthwhile to venture into this form of scientific funding.

## References

[1] Wheat RE, Wang Y, Byrnes JE, Ranganathan J. Raising money for scientific research through crowdfunding. Trends in ecology & evolution. 2013;28(2):71–2. 10.1016/j.tree.2012.11.001 . 23219380

[2] Ioannidis JP. More time for research: fund people not projects. Nature. 2011;477(7366):529–31. 10.1038/477529a . 21956312

[3] Cameron P, Corne DW, Mason CE, Rosenfeld J. Crowdfunding genomics and bioinformatics. Genome Biol. 2013;14(9):134 10.1186/gb-2013-14-9-134 24079746 PMC4054678

[4] Perlstein EO. Anatomy of the Crowd4Discovery crowdfunding campaign. SpringerPlus. 2013;2:560 10.1186/2193-1801-2-560 24255854 PMC3824701

[5] Byrnes JE, Ranganathan J, Walker BL, Faulkes Z. To Crowdfund Research, Scientists Must Build an Audience for Their Work. PLoS ONE. 2014;9(12):e110329 10.1371/journal.pone.0110329 25494306 PMC4262210

[6] Faulkes Z. #SciFund round 4 analysis. 2014. http://scifundchallenge.org/blog/2014/03/18/scifund-round-4-analysis/.

[7] Li FW, Pryer KM. Crowdfunding the Azolla fern genome project: a grassroots approach. GigaScience. 2014;3:16 10.1186/2047-217X-3-16 25276348 PMC4178311

[8] Dragojlovic N, Lynd LD. Crowdfunding drug development: the state of play in oncology and rare diseases. Drug discovery today. 2014;19(11):1775–80. 10.1016/j.drudis.2014.06.019 . 24973645

[9] Hui J GE, Gergle D. Understanding and leveraging social networks for crowdfunding: implications for support tools. Proceedings of ACM. 2014:6.

[10] Planetary Resources. ARKYD: a space telescope for everyone. 2013. https://www.kickstarter.com/projects/arkydforeveryone/arkyd-a-space-telescope-for-everyone-0/.

[11] Vallabh S, Minikel E. Can anle138b delay the onset of genetic prion disease? 2013. https://experiment.com/projects/can-anle138b-delay-the-onset-of-genetic-prion-disease/.

[12] The uBiome Team. uBiome–sequencing your microbiome. 2013. https://www.indiegogo.com/projects/ubiome-sequencing-your-microbiome/.

[13] Byrnes J. How much can you get with science crowdfunding? How about ONE MILLION DOLLARS! 2013. http://scifundchallenge.org/blog/2013/06/27/how-much-can-you-get-with-science-crowdfunding-how-about-one-million-dollars/.

[14] Experiment. Project success illustrated through social proof. 2014 http://blog.experiment.com/post/102283952627/project-success-illustrated-through-social-proof/.

[15] Human Food Project. American Gut–what’s in your gut? 2013. https://www.indiegogo.com/projects/american-gut-what-s-in-your-gut—7/.

[16] OpenWorm. OpenWorm: a digital organism in your browser. 2014. https://www.kickstarter.com/projects/openworm/openworm-a-digital-organism-in-your-browser/.

[17] OpenTrons. OpenTrons: open-source rapid prototyping for biology. 2014. https://www.kickstarter.com/projects/932664050/opentrons-open-source-rapid-prototyping-for-biolog/.

[18] Adapteva. Parallella: a supercomputer for everyone. 2012. https://www.kickstarter.com/projects/adapteva/parallella-a-supercomputer-for-everyone/.

[19] Experiment. Learning from failure. 2014. http://experiment.com/blog/94-learning-from-failures/.

